# A Risk Model for Predicting Fetuses with Trisomy 21 Using Alpha-Fetoprotein Variants L2 Combined with Maternal Serum Biomarkers in Early Pregnancy

**DOI:** 10.1007/s43032-021-00762-5

**Published:** 2021-11-08

**Authors:** Yiming Chen, Bin Wu, Yijie Chen, Wenwen Ning, Huimin Zhang

**Affiliations:** 1 Department of Prenatal Diagnosis and Screening Center, Hangzhou Women’s Hospital (Hangzhou Maternity and Child Health Care Hospital), No. 369, Kunpeng Road, Shangcheng District, Hangzhou, Zhejiang 310008, China; 2 The Fourth School of Clinical Medicine, Zhejiang Chinese Medical University, Hangzhou, Zhejiang 310053 China

**Keywords:** First trimester, Alpha fetoprotein variants L2, Trisomy 21, Risk model establishment, Diagnostic value, Retrospective case–control study

## Abstract

To establish a risk prediction model and the clinical value of trisomy 21 using alpha-fetoprotein variants L2 (AFP-L2) combined with maternal serum biomarkers and nuchal translucency (NT) thickness in early pregnancy. A retrospective case–control study was conducted. The subjects were divided into the case group (*n* = 40) or the control group (*n* = 40). An enzyme-linked immunosorbent assay was used to measure the maternal serum AFP-L2 level in both groups. The AFP-L2 single-index or multi-index combined risk model was used to predict the efficiency of trisomy 21. The best cut-off value and area under the curve (AUC) were determined to evaluate the predictive efficacy of different risk models constructed by AFP-L2. The maternal serum AFP-L2 level in the case group was 1.59 (0.61–3.61) Multiple of medium (MoM), which was higher than 1.00 (0.39–2.12) MoM in the control group (*P* < 0.001). The free beta-human chorionic gonadotropin (free β-hCG) level and NT in the case group were significantly higher than those in the control group (*P* < 0.001). The pregnancy-associated plasma protein A (PAPP-A) level in the case group was lower than that in the control group (*P* < 0.001). The AUC of AFP-L2 in predicting trisomy 21 was 0.797. After considering the maternal serum AFP-L2 level, the AUC, detection rate (DR), positive predictive value (PPV), negative predictive value (NPV), falsepositive rate (FPR), false negative rate (FNR), positive likelihood ratio (+LR), and negative likelihood ratio (-LR) were significantly improved. In this study, PAPP-A + free β-hCG + NT + AFP-L2 and PAPP-A + free β-hCG + AFP-L2 increased the integrated discrimination improvement (IDI) and net classification improvement (NRI) of predicting fetuses with trisomy 21 (1.10% and 5.27%; 11.07% and 2.78%)  (1.10% and 5.27%; 11.07% and 2.78%), respectively, after considering the maternal serum AFP-L2 level. The maternal serum AFP-L2 level in early pregnancy had high sensitivity and specificity, and it was a good biomarker to predict fetuses with trisomy 21.

## Background

By electrophoresis, alpha-fetoprotein (AFP) variants are found in the serum after AFP binds to lensculinaris agglutinin (LCA). These variants are AFP-L1, AFP-L2, and AFP-L3 [1], with AFP-L2 weakly binding to LCA and playing an important role in monitoring the recurrence of pelvic malignant tumor during pregnancy [2].

Chromosomal aneuploidy, such as trisomy 21, trisomy 18, and trisomy 13, is the most common chromosomal abnormality in fetuses. Among these, trisomy 21, which is also known as Down’s syndrome, is the most common genetic chromosomal abnormality in newborns with birth defects. It is a syndrome characterized by irreversible mental retardation due to an additional chromosome 21, in which individuals with trisomy 21 are unable to take care of themselves. It is the most common hereditary cause of mental disability, accounting for 90% of the total number of cases with neonatal chromosomal defects. The incidence of trisomy 21 in newborns is approximately 1% [3].

In light of the fact that chromosomal abnormalities are untreatable, the burden of families and societies in caring for such individuals is enormous. Therefore, it is important to screen fetuses for trisomy 21. Presently, serum levels of AFP and free beta-human chorionic gonadotropin (free β-hCG), unconjugated estriol (uE3) or pregnancy-associated plasma protein A (PAPP-A), nuchal translucency (NT) thickness, and other specific biomarkers are combined with gestational week, day of last menstruation, weight, age, and the risk of trisomy 21 data using analytical software for prenatal screening [4–6]. Further prenatal testing is recommended for those at high risk of trisomy 21. However, it is still controversial whether an increased maternal serum AFP level is a reliable indicator of trisomy 21 in early pregnancy. For instance, studies have reported that an increased AFP level did not significantly improve the detection rate (DR) of trisomy 21 [7, 8], whereas other studies have reported opposite findings [9, 10].

There are studies reporting the prediction of fetuses with trisomy 21 using the maternal serum AFP-L2 level in the second trimester of pregnancy [11, 12], but there is no study on the construction of a risk model for predicting trisomy 21 using the maternal serum AFP-L2 level in the first trimester. Compared with the results of screening in the second trimester of pregnancy, prenatal screening in the first trimester has the advantages of an early decision-making window for clinical intervention. Moreover, the false-positive rate (FPR) of early pregnancy screening is low, which can reduce the rate of unnecessary invasive tests for all pregnant women [13]. Therefore, a retrospective case–control study was conducted to investigate the relationship between the maternal serum AFP-L2 level and trisomy 21.

## Research Objects and Methods

### Screening Objects

A retrospective case–control study was conducted to analyze the data of singleton pregnant women at 11–13^+6^ weeks of gestation visiting the Prenatal Screening Center of Hangzhou Women’s Hospital (Hangzhou Maternity and Child Health Care Hospital) from October 2015 to September 2019. The subjects were divided into the case group (*n* = 40) and the control group (*n* = 40) according to the presence or absence of trisomy 21, respectively. Women carrying fetuses with trisomy 21 were confirmed by amniotic fluid cell karyotype analysis. Women carrying normal fetuses were randomly selected using a ratio of 1:1. This study was approved by the ethics committee of Hangzhou Women’s Hospital, with the approval number of [2021] Medical Ethics Review A (3)—02.

### Diagnostic and Exclusion Criteria

#### Diagnosis Was Based on Diagnostic Criteria Established by the China Birth Defect Monitoring Network [14]

The main characteristics of trisomy 21 are wide eye distance (> 2.5 cm), collapsed nose, lateral canthus oblique, low muscle tone throughout the body, penetrating hands and/or palm high trigeminal t, short little finger and/or short or absent middle segment, I, II wide toe pitch (straw-shoe feet), tibial arch pattern on the ball of the hallux, and lower ears. Karyotype 47, XX (or XY) + 21 was the most common anomaly, accounting for 94–95% of cases. Approximately 1–2% of cases were of the trisomy 21 mosaic type, and 2–3% of cases were of the translocation type, which included D/G and G/G translocations. Cases with at least five of the aforementioned characteristics, combined with other features, were designated as trisomy 21. The final diagnosis required further chromosomal tests.

#### To Reduce the Interference of Some Factors in the Detection of the Maternal Serum AFP-L2 Level, the Following Criteria Were Excluded

(1) Twin and multiple pregnancies; (2) the presence of medical conditions such as insulin-dependent diabetes mellitus and serious pregnancy complications; (3) a history of smoking; (4) test tube baby; (5) the presence of trisomy 18, trisomy 13, or other birth defects; (6) incomplete information; and (7) discrepancies between data and serum specimens of pregnant women.

### Methods

#### Reagents and Instruments

We used the 1235 automatic time-resolved fluoroimmunoassay (DELFIA®) immunoassay analyzer ( Perkin Elmer, Shelton, USA) and matching kits (PAPP-A, free β-hCG), enhancement solution and washing reagents, quality control samples, and calibrating samples. Additionally, we used the RT-6100 microplate reader (Rayto, Shenzhen, China), 988 plate washer (Tianshi, Beijing, China), AFP-L2 reagent (BIM, San Francisco, CA, USA), and the Voluson E8 ultrasonic instrument (GE, Boston, MA, USA).

#### Detection Method

Fasting peripheral venous blood (2–3 mL) was collected within 1 week of the initial examination. Approximately 30 min after blood collection, specimens were centrifuged and stored at 2–8 °C. Maternal serum PAPP-A and free β-hCG levels were measured. The DELFIA method was used as previously described (reference). Remaining serum specimens were stored at − 80 °C. Before measurement, data of case and control groups were matched with stored serum specimens, and serum specimens were centrifuged mixed. The maternal serum AFP-L2 level was measured by a double-antibody one-step enzyme linked immunosorbent assay (ELISA).

#### Fetal NT Thickness Measurement

At 11–13^+6^ weeks of gestation, the fetal NT thickness was measured by experienced ultrasound examiners according to the standards of the Fetal Medicine Foundation (https://fetalmedicine.org/education/the-11-13-weeks-scan).

### Multiple of Medium (MoM) Was Used to Represent the Maternal Serum Levels of PAPP-A, Free β-hCG, and AFP-L2 [15, 16]

Definition and calculation formula of MoM value:

1-1 $$MoM=\frac{Original\;Conj.}{Median}$$

where Original Conj. is the original concentration of PAPP-A, free β-hCG, and AFP-L2 and median represents the median of the original concentration of the corresponding indicator [15].

In order to reduce the deviation caused by different gestational age and maternal weight, we calibrated the MoM value of each index and replaced the original concentration value with MoM value.

The MoM values were calibrated by the median equation of gestational age and the median equation of maternal weight. Taking AFP-L2 as an example, the calculation was as follows [16]:

1-2 $$GA\;Med = 10^{(-261.9+8.09 \times GA-0.09277 \times GA^{2} + 0.0004694 \times GA^{3} - 0.0000008842 \times GA^{4})}$$

[GA: gestational age (days); Med: Medium]

**Fig. 1:**
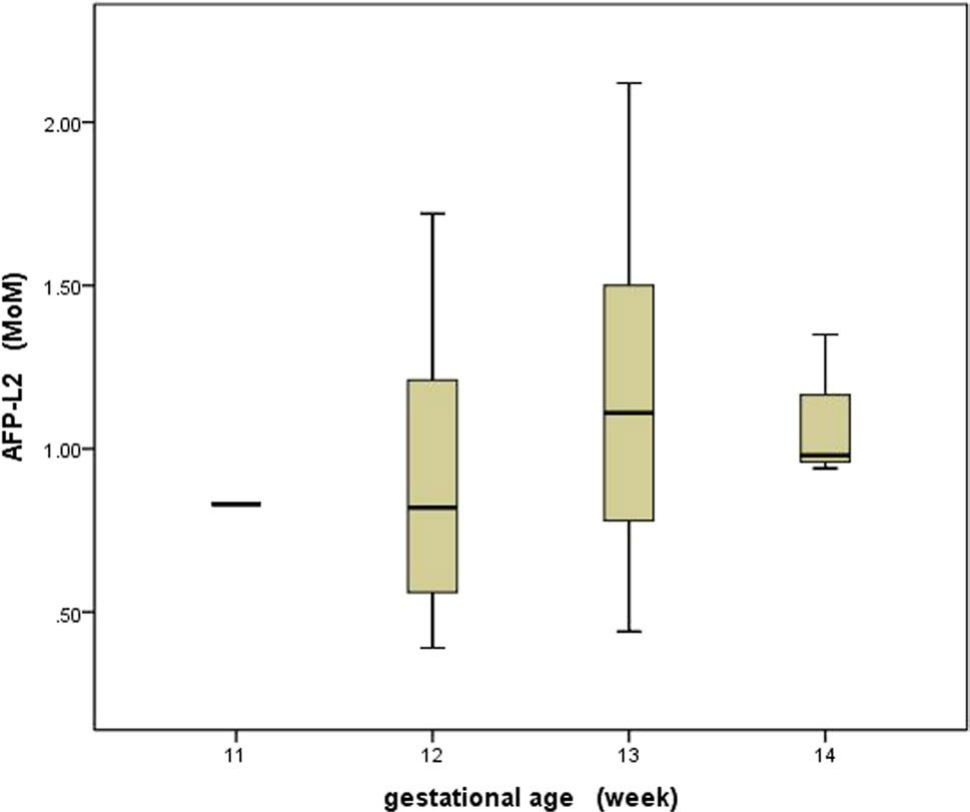
The distribution of different gestational ages of serum AFP-L2 MoM among 11–14 weeks in 40 normal control groups

The distribution of different gestational ages in the 40 normal control groups at 11–14 weeks is as follows: 0.83 (0.83–0.83) MoM, 0.82 (0.39–0.82) MoM, 1.11 (0.44–2.11) MoM, and 0.98 (0.94–0.98) MoM. There is no statistically significant difference between different gestational ages (*x*^*2*^ = 1.861, *P* = 0.602) in Fig. 1.

1-3 $$Maternal\;Weight\;Med = 0.8852 - \frac{9.465}{{maternal\;weight}}$$

Similarly, PAPP-A, free β- hCG, NT and AFP-L2 so on. According to the above 1–2 formula and 1–3 formula median equation, the MoM value is adjusted as shown in 1–4 formula, and the adjusted MoM value is used for modeling calculation [15].

1-4 $$Adjusted\:MoM = \frac{MoM}{{GA\:Med \times Weight\:Med}}$$

### Methods for Establishing Different Risk Prediction Models

#### The Risk Value of Maternal Age Was Calculated as Follows [17]:

1-5 $$\mathrm r\mathrm i\mathrm s{\mathrm k}_{\mathrm m\mathrm a\mathrm t\mathrm e\mathrm r\mathrm n\mathrm a\mathrm l\;\mathrm a\mathrm g\mathrm e} = 0.0000697+\exp^{-18.4367+0.286\times(\mathrm m\mathrm a\mathrm t\mathrm e\mathrm r\mathrm n\mathrm a\mathrm l\;\mathrm a\mathrm g\mathrm e-0.5)}$$

#### In this Scheme, the Probability Density Function of Normal Distribution Was Used to Calculate the Sample Likelihood Ratio, and the Results Were Used as the Risk Prediction Score of Fetuses with Trisomy 21

The likelihood ratio was calculated as follows [16, 18]:

Likelihood ratio:

1-6 $$LR\; {multinorm} = \frac{\mathrm{likelihood\;of\;Trisomy}\;21}{\mathrm{likelihood\;of\;controls}}$$

#### The Ultimate Risk of Trisomy 21 Was Calculated as Follows

1-7 $$risk_{\mathrm {Trisomy}\,21} = \frac{1}{{LR\; {multinorm}} \times Risk\;{\mathrm{maternal\;age}}}$$

#### Fifteen Models Were Constructed by the Above Steps

PAPP-A MoM, free β- hCG MoM, NT MoM, and AFP-L2 MoM were singly linked; PAPP-A + free β- hCG, PAPP-A + NT, PAPP-A + AFP-L2, free β- hCG + NT, free β- hCG + AFP-L2 and NT + AFP-L2. PAPP-A + free β- hCG + NT, PAPP-A + free β- hCG + AFP-L2, PAPP-A + NT + AFP-L2, free β- hCG + NT + AFP-L2, PAPP-A + free β- hCG + NT + AFP-L2.

### Statistical Analysis

Excel 2007 software was used to establish a database of test results, and IBM SPSS 21.0 software (IBM-SPSS, Chicago, IL, USA) was used for statistical analysis. Measurement data were tested for normality, and data presenting skewed distribution were expressed as medians and percentiles [M (P_2.5_, P_97.5_)]. Comparisons between the two groups were performed using Mann–Whitney U test. Additionally, Python 3.8 (https://www.python.org/), a multivariate normal probability model, was incorporated based on Bayes’ theorem [18]. The receiver operating characteristic (ROC) curve, area under the curve (AUC), integrated discrimination improvement (IDI), and net classification improvement (NRI) [19] were used to evaluate the performance of AFP-L2 and other biomarkers in building a risk model to predict fetuses with trisomy 21. *P* < 0.05 was considered statistically significant.

## Results

### Baseline Demographic Data of Pregnant Women in Both Groups

The maternal age in the case group was higher than that in the control group, and the difference was statistically significant (*Z* = 2.213, *P* = 0.027). The gestational age in the case group was lower than that in the control group, and the difference was not statistically significant (*Z* = 1.697, *P* = 0.090). The maternal weight in the case group was greater than that in the control group, and the difference was statistically significant (*Z* = 2.147, *P* = 0.032), as shown in Table 1.

**Table 1:** Basic demographic information of the pregnant women in the trisomy 21 and control groups

Group	n	Maternal age (years)	Maternal weight (kg)	Gestational age (days)
Control	40	28.44 (20.35–33.86)	50.10 (42.90–63.00)	89.00 (80.05–97.00)
Trisomy21	40	29.92 (24.11–37.79)	53.30 (39.13–74.73)	87.00 (69.10–96.98)
*Z*		2.213	2.147	1.697
*P*		0.027^**^	0.032^**^	0.090

^Data are presented as median (P2.5–P97.5); **^P < 0.05

### Comparison of Maternal Serum AFP-L2, PAPP-A, and Free β-hCG Levels, As well as Fetal NT Thickness, Between the Two Groups

The maternal serum AFP-L2 level in pregnant women in the case group was 1.59 (0.61–3.61) MoM, which was higher than that in the control group at 1.00 (0.39–2.12) MoM, and the difference was statistically significant (*P* < 0.001). The free β-hCG level and NT thickness in the case group were higher than those in the control group, and the differences were statistically significant (*P* < 0.001). The maternal serum PAPP-A level in the case group was lower than that in the control group, and the difference was statistically significant (*P* < 0.001), as shown in Table 2.

**Table 2:** Comparison of MoM levels of serum AFP-L2 and other screening markers during early pregnancy between trisomy 21 and the xontrol group

Group	n	PAPP-A	free β- hCG	NT	AFP-L2
Control	40	0.88 (0.20–3.74)	1.02 (0.33–3.98)	0.87 (0.59–1.15)	1.00 (0.39–2.12)
Trisomy 21	40	0.37 (0.04–1.65)	1.76 (0.15–6.95)	1.17 (0.59–2.10)	1.59 (0.61–3.61)
*Z*		5.192	3.675	4.197	4.566
*P*		< 0.001^*^	< 0.001^*^	< 0.001^*^	< 0.001^*^

*PAPP-A* pregnancy-associated plasma protein A, *free β- hCG* free beta-subunit of human chorionic gonadotropin, *NT* nuchal transparency, *AFP-L2* alpha-fetoprotein variants L2, *MoM* multiple of the median. Data are presented as median (P_2.5_–P_97.5_); ^*^P < 0.001

### Diagnostic Value of Maternal Serum AFP-L2, PAPP-A, and Free β-hCG Levels, As well as Fetal NT Thickness, in Predicting Fetuses with Trisomy 21 in Early Pregnancy

**Fig. 2:**
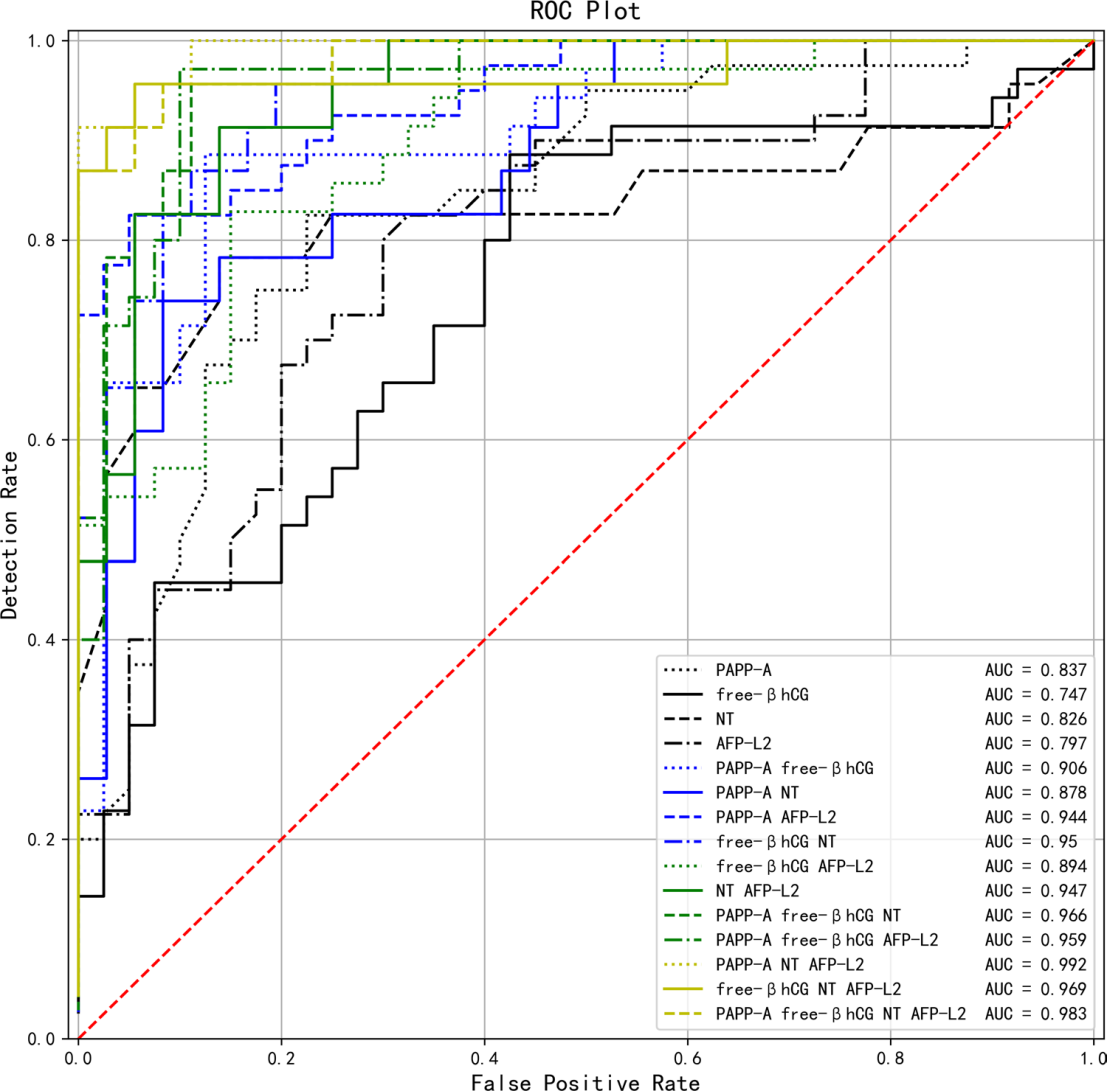
ROC curves of trisomy 21 predicted by the combination of single or multiple indicators of PAPP-A, free β- hCG, NT, and AFP-L2

The AFP-L2 predicted AUC of fetuses with trisomy 21 was 0.797 (95%CI: 0.601–948, *P* = 0.009). According to the ROC curve, the optimal cut-off value of AFP-L2 for predicting fetuses with trisomy 21 was 1.234 MoM, and the sensitivity and specificity were 0.805 and 0.695, respectively. The AUC of the single index model was PAPP-A > NT > AFP-L2 > free β-hCG. The top five AUC values of the multi-indicator model were PAPP-A + NT + AFP-L2 > PAPP-A + free β-hCG + NT + AFP-L2 > free β-hCG + NT + AFP-L2 > PAPP-A + free β-hCG + NT  > PAPP-A + free β-hCG + AFP-L2, as shown in Table 3 and Fig. 2.

**Table 3:** The diagnostic value of single index or multiple index combination of different markers in predicting Trisomy 21

Screening method or model	Youden index		Sensitivity	Specificity	cut-off	AUC	95%CI	*P*
PAPP-A		0.600	0.825	0.775	0.655	0.837	0.727–0.981	0.001^**^
free β- hCG		0.461	0.886	0.575	1.095	0.747	0.499–0.872	0.077
NT		0.644	0.783	0.861	1.035	0.826	0.658–0.955	0.004^**^
AFP-L2		0.500	0.805	0.695	1.234	0.797	0.601–0.948	0.009^**^
PAPP-A + free β- hCG		0.761	0.886	0.875	1/1042	0.906	0.718–0.994	0.001^**^
PAPP-A + NT		0.656	0.739	0.917	1/485	0.878	0.821–1.000	< 0.001^*^
PAPP-A + AFP-L2		0.775	0.825	0.950	1/438	0.944	0.863–1.000	< 0.001^*^
free β- hCG + NT		0.762	0.957	0.806	1/1378	0.950	0.846–1.000	< 0.001^*^
free β- hCG + AFP-L2		0.679	0.829	0.850	1/868	0.894	0.680–0.964	0.002^**^
NT + AFP-L2		0.774	0.913	0.861	1/994	0.947	0.799–1.000	< 0.001^*^
PAPP-A + free β- hCG + NT		0.845	0.957	0.889	1/1318	0.966	0.845–1.000	< 0.001^*^
PAPP-A + free β- hCG + AFP-L2		0.871	0.971	0.900	1/1033	0.959	0.812–1.000	< 0.001^*^
PAPP-A + NT + AFP-L2		0.913	0.913	1.000	1/592	0.992	0.954–1.000	< 0.001^*^
free β- hCG + NT + AFP-L2		0.901	0.957	0.944	1/716	0.969	0.894–1.000	< 0.001^*^
PAPP-A + free β- hCG + NT + AFP-L2		0.873	0.957	0.917	1/1071	0.983	0.922–1.000	< 0.001^*^

*PAPP-A* pregnancy-associated plasma protein A, *free β- hCG* free beta-subunit of human chorionic gonadotropin, *NT* nuchal transparency, *AFP-L2* alpha fetoprotein variants L2, *AUC* area under curve, *CI* confidence interval. ^*^P < 0.001, ^**^P < 0.05

### Comparison of Single- and Multi-index Models of AFP-L2, PAPP-A, Free β-hCG, and Fetal NT Thickness in Predicting Fetuses with Trisomy 21

Table 4 shows that the DR, positive predictive value (PPV), negative predictive value (NPV), FPR, false negative rate (FNR), positive likelihood ratio (+ LR), and negative likelihood ratio (-LR) of PAPP-A + free β-hCG + NT + AFP-L2 and PAPP-A + free β-hCG + AFP-L2 were all superior to those of PAPP-A + free β-hCG + NT and PAPP-A + free β-hCG. Table 5 shows that PAPP-A + free β-hCG + NT + AFP-L2 and PAPP-A + free β-hCG + AFP-L2 increased the IDI and NRI of predicting fetuses with trisomy 21 by 1.10% and 5.27% and 11.07% and 2.78%, respectively, after considering the maternal serum AFP-L2 level.

**Table 4:** Predicting the diagnostic effect of Trisomy 21 by single index or multiple index combination of different markers

Screening method or model	DR	PPV	NPV	FPR	FNR	+ LR	-LR
PAPP-A	0.800	0.786	0.816	0.225	0.175	3.667	0.226
free β- hCG	0.720	0.646	0.852	0.425	0.114	2.084	0.199
NT	0.831	0.783	0.861	0.139	0.217	5.635	0.252
AFP-L2	0.750	0.727	0.778	0.300	0.200	2.667	0.286
PAPP-A + free β- hCG	0.880	0.861	0.897	0.125	0.114	7.086	0.131
PAPP-A + NT	0.847	0.850	0.846	0.083	0.261	8.870	0.285
PAPP-A + AFP-L2	0.888	0.943	0.844	0.050	0.175	16.500	0.184
free β- hCG + NT	0.864	0.759	0.967	0.194	0.043	4.919	0.054
free β- hCG + AFP-L2	0.840	0.829	0.850	0.150	0.171	5.524	0.202
NT + AFP-L2	0.881	0.808	0.939	0.139	0.087	6.574	0.101
PAPP-A + free β- hCG + NT	0.915	0.846	0.970	0.111	0.043	8.609	0.049
PAPP-A + free β- hCG + AFP-L2	0.933	0.895	0.973	0.100	0.029	9.714	0.032
PAPP-A + NT + AFP-L2	0.966	1.000	0.947	0.000	0.087	-	0.087
free β- hCG + NT + AFP-L2	0.949	0.917	0.971	0.056	0.043	17.217	0.046
PAPP-A + free β- hCG + NT + AFP-L2	0.932	0.880	0.971	0.083	0.043	11.478	0.047

*PAPP-A* pregnancy-associated plasma protein A, *free β- hCG* free beta-subunit of human chorionic gonadotropin, *NT* nuchal transparency, *AFP-L2* alpha fetoprotein variants L2, *DR* detection rate, *FPR* false-positive rate, *FNR* false-negative rate, *PPV* positive predictive value, *NPV* negative predictive value, + *LR* positive likelihood ratio*, − LR* negative likelihood ratio

**Table 5:** AFP-L2 combined with free β- hCG and other markers were used to improve the predicted

Model 1	Model 2	IDI (%)	*P* value for IDI	NRI (%)	*P* value for NRI
PAPP-A	PAPP-A AFP-L2	10.67	< 0.001^*^	17.50	0.026^**^
free β-hCG	free β-hCG AFP-L2	12.57	< 0.001^*^	21.79	0.021^**^
NT	NT AFP-L2	1.71	0.360	13.04	0.160
PAPP-A free β-hCG	PAPP-A free β-hCG AFP-L2	1.10	0.319	11.07	0.046^**^
PAPP-A NT	PAPP-A NT AFP-L2	9.49	< 0.001^*^	25.72	0.005^**^
free β-hCG NT	free β-hCG NT AFP-L2	5.41	0.014^**^	13.89	0.056
PAPP-A free β-hCG NT	PAPP-A free β-hCG NT AFP-L2	5.27	0.001^**^	2.78	0.159

*PAPP-A* pregnancy-associated plasma protein A, *free β- hCG* free beta-subunit of human chorionic gonadotropin, *NT* nuchal transparency, *AFP-L2* alpha fetoprotein variants L2, *IDI* integrated discrimination improvement, *NRI* net reclassification improvement; ^*^P < 0.001, ^**^P < 0.05

## Discussion

Prenatal screening involves screening of the maternal age, followed by more in-depth screening during mid- and early pregnancy. The combined detection of aneuploidy biomarkers, such as PAPP-A, free β-hCG, and the fetal NT thickness, in early pregnancy (11–13 weeks^+6^), is widely used in prenatal screening of fetuses with trisomy 21 or trisomy 18 [20, 21]. When the false positive rate is 5%, this screening method can detect 62% of fetuses with trisomy 21 [22], indicating that 38% of fetuses with trisomy 21 will be missed. To improve the detection rate of Down’s syndrome and to reduce subsequent medical disputes, it is important to identify new prenatal screening biomarkers and screening approaches. In this study, we examined 40 cases of trisomy 21 and a corresponding number of controls. After combining the maternal serum AFP-L2 level with other serum biomarkers (PAPP-A + free β-hCG) and the fetal NT thickness in early pregnancy, the prediction efficacy of different risk models constructed by AFP-L2 was compared using AUC, + LR, -LR, IDI, and NRI.

The results showed that the serum AFP-L2 level in pregnant women carrying fetuses with trisomy 21 was higher than that in women carrying normal fetuses during early pregnancy, and the differences were statistically significant (*P* < 0.001). There is no study reporting serum AFP-L2 levels in pregnant women with trisomy 21 in early pregnancy, although there are studies reporting serum AFP-L2 levels in pregnant women with trisomy 21 in middle pregnancy [11, 12, 23]. These studies have revealed that the maternal serum AFP-L2 level in pregnant women with trisomy 21 increased, and the differences were statistically significant (*P* < 0.05), similar to the results of this study. Newby et al. [24] reported that the placental AFP level in pregnant women carrying fetuses with trisomy 21 was significantly increased, whereas the hepatic AFP level was unchanged. Additionally, the maternal serum AFP level was decreased, which may be associated with specific AFP transport defects. Yamamoto et al. [25] also confirmed that the placental AFP-L3 level in pregnant women carrying fetuses with trisomy 21 was elevated, which may explain the increased serum level of AFP-L3 in these women. Therefore, we speculate that the increased serum level of AFP-L2 in pregnant women carrying fetuses with trisomy 21 in early pregnancy is similar to that of AFP or AFP-L3. Additionally, there may also be a specific AFP transport defect, resulting in the increased maternal serum level of AFP-L2.

We previously reported that the AUC of AFP-L2 in the screening of fetuses with Down’s syndrome in the second trimester of pregnancy was 0.891 [12]. Yamamoto et al. [23] showed that the AUC of AFP-L3 and AFP MoM in the maternal serum was 0.835 and 0.700, respectively, and no association was found between AFP-L3 and AFP MoM (*r* = 50.006). Yamamoto et al. [26] also measured the AFP level and AFP-L3% in the maternal serum in the second trimester of pregnancy, and the AUCs of AFP MoM, AFP-L3%, AFP-L3 MoM, and AFP-L3 MoM/AFP MoM were 0.750, 0.868, 0.949, and 0.946, respectively. Following analysis, it was suggested that AFP-L3 MoM should replace AFP-L3% in the screening of fetuses with trisomy 21. Bredaki et al. [27] also confirmed that after adjusting the maternal characteristics and medical history variables affecting measurements, the fitted risk model should be expressed using the maternal serum AFP MoM in early pregnancy. To reduce the deviations caused by differences in gestational age and maternal weight, we calibrated the MoM value of each index and replaced the original concentration value with the MoM value. The results in Table 3 showed that the AUC of trisomy 21 predicted by the AFP-L2 MoM in early pregnancy was 0.797, which was slightly lower than the AUC of mid-pregnancy previously reported [12, 23, 26]. It remains to be determined whether there is any association in the relatively lower AFP-L2 concentration between early and mid-pregnancy.

This study also showed that PAPP-A + NT + AFP-L2 and PAPP-A + free β-hCG + NT + AFP-L2 were the best risk models of AFP-L2 when combined with other biomarkers in early pregnancy. Tables 4 and 5 also show that the combined use of other biomarkers in early pregnancy could improve the predictive value of trisomy 21. In a previous study, AFP-L2 + AFP-L3, when combined with biomarkers of mid-pregnancy, were compared using different models, and the results confirmed that combined screening was superior to single screening [12], similar to the results of this study.

As shown in Table 1, maternal age and maternal weight loss in the case group were significantly higher than those in the control group (all *P* < 0.05), consistent with previous studies. Snijders et al. [28] reported that the risk of trisomy 21 increased with increasing maternal age. Our previous retrospective study also confirmed that the incidence of trisomy 21 was lowest in pregnant women younger than 25 years old (1.66/million) and highest in pregnant women older than 40 years old (45.56/million), and the difference was statistically significant (*P* < 0.001) [29]. Similarly, Hildebrand et al. [30] showed that maternal obesity increased the risk of fetuses with trisomy 21, similar to the results of this study.

In general, the construction of risk models requires a large cohort. As the number of subjects in the case group and the control group in this study was 40 per group, there may be some bias, which should be assessed in further retrospective or prospective studies with larger cohorts. This is the main limitation of this study.

## Conclusions

In conclusion, the maternal serum AFP-L2 level in early pregnancy had high sensitivity and specificity, indicating that it was a good biomarker to predict fetuses with trisomy 21. The maternal serum AFP-L2 level, when combined with other biomarkers, in early pregnancy improved the predictive value of trisomy 21.

## Data Availability

All data generated or analyzed during this study are included in the supplementary file and this published article.

## Data Availability Statement

Data sharing is not applicable to this article as no new data were created or analyzed in this study.

## Abbreviations

AFP: Alpha-fetoprotein
AFP-L2: Alpha-fetoprotein variants L2
PAPP-A: Pregnancy-associated plasma protein A
Free β-hCG: Free beta human chorionic gonadotropin
NT: Fetal neck transparent layer thickness
IDI: Integrated discrimination improvement
NRI: Net reclassification index or improvement
MoM: Multiple of the median
ROC: Receiver operating characteristic
AUC: Area under curve
FNR: False negative rate
FPR: False positive rate
PPV: Positive predictive value
NPV: Negative predictive value
+ LR: Positive likelihood ratio
− LR: Negative likelihood ratio
ELISA: Enzyme linked immunosorbent assay
